# Arthrocentesis and Sodium Hyaluronate Infiltration in Temporomandibular Disorders Treatment. Clinical and MRI Evaluation

**DOI:** 10.3390/jfmk5010018

**Published:** 2020-03-06

**Authors:** Mario Santagata, Roberto De Luca, Giorgio Lo Giudice, Antonio Troiano, Giuseppe Lo Giudice, Giovanni Corvo, Gianpaolo Tartaro

**Affiliations:** 1 Multidisciplinary Department of Medical-Surgical and Dental Specialities, Oral and Maxillofacial Surgery Unit, AOU University of Campania “Luigi Vanvitelli”, 80138 Naples, Italy; mario.santagata@unicampania.it (M.S.); gianpaolotartaro@unicampania.it (G.T.); 2 Maxillofacial Surgery Unit, Department of Neurosciences, Reproductive and Odontostomatological Sciences, University of Naples "Federico II”, 80138 Naples, Italy; robertodeluca89@yahoo.it (R.D.L.); antoniotroiano85@gmail.com (A.T.); 3 Department of Biomedical and Dental Sciences and Morphofunctional Imaging, Messina University, 98122 Messina, Italy; logiudiceg@unime.it

**Keywords:** TMJ, temporomandibular joint disorders, arthrocentesis, sodium hyaluronate, hyaluronic acid

## Abstract

Arthrocentesis in temporomandibular joint disorders can be associated with the intra-articular infiltration of various drugs with the objective of increase treatment efficacy. The aim of this study was to evaluate the clinical indexes variation in patients affected by temporomandibular joint disorders treated with arthrocentesis and sodium hyaluronate (SH) injections. A total of 28 patients suffering from temporomandibular joint disorders underwent one cycle of five arthrocentesis and infiltrations of sodium hyaluronate. Spontaneous mouth opening improved from 36.3 ± 7.5 mm to 45.1 ± 1.9 mm at six months follow-up. A significant reduction in the pain at rest and during mastication mean values emerged at follow-up (*p* < 0.0001). The mean masticatory efficiency, evaluated through a visual analogic scale, showed improvement at the follow-up period, highlighted by the increase of mean value from a baseline of 3.1 ± 1.2 to a mean value of 8.5 ± 1.2 (*p* < 0.0001). The mean severity of the joint damage at baseline time was 2.4 ± 0.9 and decreased to 0.4 ± 0.3 at the end of the follow-up period. The decrease in values is confirmed by statistical test (*p* < 0.05). Our data show how arthrocentesis integrated with sodium hyaluronate infiltrations performed under local anesthesia is a valid method of treating temporomandibular joint disorders.

## 1. Introduction

Temporomandibular joint (TMJ) disorders include disc displacement and degenerative and/or inflammatory pathologies. The TMJ accounts for about 10% of the population with a predilection for female sex and is often associated with chronic pain and limited function resulting in decreased quality of life for the patient [1,2,3,4].

Different conservative and surgical treatments have been studied to restore stomatognathic function and improve clinical symptoms [5,6,7]. Conservative treatments include behavioral therapy, administration of non-steroidal anti-inflammatory drugs and corticosteroids, bite splints, botulinum toxin injections, and physical therapy [8,9,10,11,12,13].

An alternative in surgical treatments is arthrocentesis: it is a minimally invasive procedure, commonly used to remove inflammatory mediators associated with nociceptive processes within the synovial fluid [14,15,16]. This procedure can be associated with the injection of various drugs, such as sodium hyaluronate (SH) with the objective of increasing treatment efficacy [17,18].

SH is a physiological component of synovial fluid in joints and performs a lubricant function. Its ability to retain water modifies the viscosity of the synovial fluid increasing the hydration, allowing mechanical shock resistance and overall cartilage stability. 

The therapeutic effect of this molecule had been exploited in orthopedics diseases reducing subchondral bone damage, chondrocyte apoptosis, cartilage inflammation, and overall cartilage deterioration. SH intra-articular infiltration in worn joints results in pain reduction and function improvement [19,20].

The aim of this study was to evaluate the clinical indexes variation in patients affected by temporomandibular joint disorders treated with arthrocentesis and SH injections.

## 2. Materials and Methods 

A total of 28 patients suffering from temporomandibular joint disorders (27 women and 1 man, age range: 16–69 years, mean age: 40,6 years) underwent a cycle of five arthrocentesis with injections (1 per week for 5 weeks) of 1 mL hyaluronic acid (Sinovial® Mini 0,8%, IBSA Farmaceutici Italia, Lodi, Italy) into both TMJs. The infiltrations were performed according to the protocol in use for degenerative knee pathology treatment [21,22,23,24].

The protocol was approved by the internal ethical committee of the University (AOU-SUN prot. 3731, 16 May 2015). Informed consents were signed before every procedure.

Inclusion criteria were disc displacement without reduction with limited opening according to the Diagnostic Criteria for Temporomandibular Disorders (DC/TMD) classification system, TMJ pain at rest or evoked by palpation or forced mouth opening, functional limitation to opening movements, failure of conservative therapy alone (non-steroidal anti-inflammatory drugs and corticosteroids), and failure of gnathological treatment with occlusal bite [25,26].

Exclusion criteria were previous surgical treatments and arthrocentesis, intra-articular infiltration of drugs, Wilkes classification < 1 and > 4, and potential risk factors of SH allergic reactions. 

All patients underwent TMJ scans with Magnetic Resonance Imaging (MRI) before treatment (T0) and at 6 months follow-up (T1) in order to assess disc disorders, condylar cartilage erosions, and condylar excursion differences. The clinical parameters collection was performed, recording the data at the time of the diagnosis (baseline) and after 6 months from the end of the treatment. 

The clinical data collected were:

- Severity of the joint damage (assessed using Wilkes staging system for TMJ internal derangement stages 0–5) [27].
- Maximum non-assisted mouth openings (in mm).
- Pain at rest and during mastication, assessed by means of a Visual Analogic Scale (VAS) from 0 to 10, with the extremes “no pain” and “pain as bad as the patient ever experienced”, respectively [28].
- Mastication efficiency, assessed by a VAS scale from 0 to 10, whose extremes were “eating only semi-liquids” and “eating solid hard food”, respectively.

The research was based on a comparison of changes in clinical parameters analyzed before and six months after the end of treatment. 

Demographic data are summarized in Table 1 (Table 1). 

**Figure 1:**
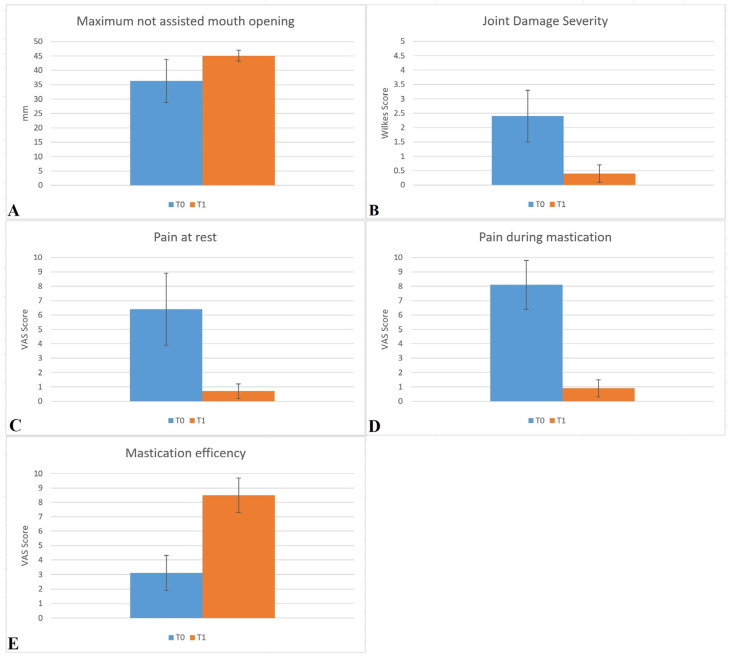
Graphical representation of collected data at baseline (T0) and 6 months follow-up (T1). **(A)** Maximum non-assisted mouth opening in mm. **(B)** Joint damage severity expressed in Wilkes Score. **(C)** Pain at rest expressed in the Visual Analogic Scale (VAS) Score. **(D)** Pain during mastication expressed in VAS Score. **(E)** Masticatory efficiency expressed in VAS Score.

Clinical parameter data at T0 and T1 are summarized in Table 2 and Figure 1. 

**Figure 2:**
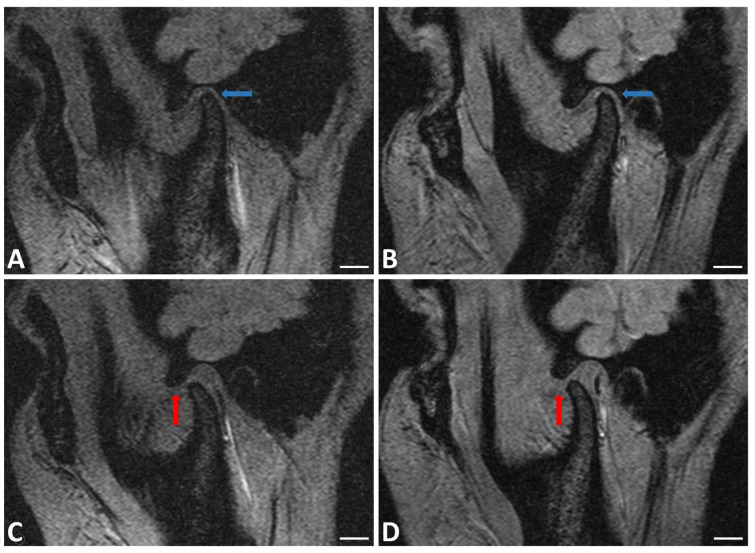
Right TMJ MRI in sagittal plane of patient with the jaw closed at T0 (**A**) and T1 (**B**) and open at T0 (**C**) and T1 (**D**). Increased disk and cartilage thickness (blue arrows) and increased excursion of mandibular condyle (red arrows) were observed. BAR = 1 cm

The instrumental analysis performed on TMJs at T0 and T1 is shown in Figure 2.

### 2.1. Clinical Procedure 

Arthrocentesis of upper joint space was performed under local anesthesia with 20 mg/mL Mepivacaine Hydrochloride (Carbocaine^®^ 2% Aspen Pharma Trading Limited, Dublin IE). The anesthetic was first injected into joint cavity, relaxing this virtual space. Subsequently, the needle was gently withdrawn to the skin surface, thus anesthetizing soft tissues over the joint. Then, the patients were instructed to keep open the mouth and a 21 gauge needle with a 10 mL syringe was inserted 1 cm anterior and 2 mm inferior along the Holmlund line (the canthal-tragal line) and oriented 30° posteriorly and inferiorly to the sagittal plane until bony contact at the medial wall of the glenoid fossa was performed. The second 21 G needle was inserted 7 mm anterior and 2 mm inferior along the Holmlund line until bony contact was performed. Then, an arthrocentesis with Ringer’s solution was carried out to eliminate catabolites in the synovial fluid. Once arthrocentesis was completed, the second needle was removed and 1 mL of SH in 3” was injected into the joint.

The same protocol was repeated for the further five infiltrations (1 per week for 5 weeks). Patients followed a physiotherapy program of guided mouth opening that went on for 4 weeks after the last injection.

### 2.2. Imaging Procedure 

All scans were carried out by a 1.5 Tesla MRI (Magnetom™ Avanto, Siemens, Erlangen, Germany).

MRI was performed using the following parameters: 1.5 mm section thickness, 256 × 256 matrix, 150 × 100 mm field of view, partial saturation pulse sequence with 34 msec TR, 10.00 msec TE in parasagittal plane and 4 mm section thickness, 256 × 256 matrix, 160 mm × 100 mm field of view with 9650 msec TR and 33 msec TE in coronal plane.

MRI images were horizontally corrected with respect to the long axis of the condyle. MRI scan before the treatment (T0) and 6 months after the last of the five infiltrations (T1) was performed.

### 2.3. Statistical Analysis

The numerical data are expressed as mean and standard deviations. Statistical analyses were performed using SPSS 17.0 for Windows package, and *t*-student test was performed and *p* < 0.05 was considered statistically significant.

The results were graphically elaborated (Figure 1). 

## 3. Results

The analysis of data showed that after the cycle of infiltrations maximum mouth opening index improved from an initial mean value of 36.3 ± 7.5 mm (T0) to 45.1 ± 1.9 mm (T1). Statistical analysis shows that the difference is statistically significant (*p* < 0.05) (Table 2, Figure 1A).

The mean severity of the joint damage at baseline time was 2.4 ± 0.9 and decreased thereafter, reaching 0.4 ± 0.3 at the end of the follow-up period. The decrease in values is confirmed by statistical test (*p* < 0.05) (Table 2, Figure 1B). 

A high significant decrease in the pain at rest parameter (*p* < 0.0001) was recorded. The mean value moved from a pretreatment mean value of 6.4 ± 2.5 to a posttreatment mean value of 0.7 ± 0.5 (Table 2, Figure 1C).

A high significant decrease in the pain at mastication values (*p* < 0.0001) was recorded. The baseline mean pain values during mastication moved from 8.1 ± 1.7 (T0) to 0.9 ± 0.6 (T1) (Table 2, Figure 1D).

The mean masticatory efficiency VAS value at baseline was 3.1 ± 1.2. At T1, the mean increase was 8.5 ± 1.2. The *p* value < 0.0001 highlights the significant difference between the two groups T0 *vs*. T1 (Table 2, Figure 1E). 

Thirteen patients (46.4%) considered “moderate” treatment tolerability, thirteen patients (46.4%) reported “good” tolerability, and two patients (7.2%) considered "slight" treatment tolerability.

MRI assessment mostly showed increased excursion of mandibular condyle, recovery from cartilage erosion and increased disk thickness; inversely, condylar and meniscal position improvement were not observed (Figure 2).

Clinical data at follow-up are summarized in Table 2.

## 4. Discussion

Temporomandibular joint disorders are typically associated with structural alterations in joint tissues, such as cartilage degradation and subchondral bone alterations. Collagenases and matrix metalloproteinase, zinc-containing proteins with enzymatic activity, likely play roles in this process. 

In patients suffering from joint disorders where disc displacement without reduction and limited opening occurs, according to DC/TMD classification, arthrocentesis with two-needle technique may be considered to carry out the lavage of the superior joint space [25,29].

This minimally invasive surgical therapy allows reducing pain, removing the biochemical causes of inflammation, and removing debris particles from the worn cartilage. 

Moreover, it exerts a mechanical effect, consenting, thanks to the endocavitary pressure raise and the alignment and/or adhesions of the disc, although the arthrocentesis results are hardly predictable [26,30].

Many studies conducted from the 1970s onward on knee osteoarthrosis demonstrated that intra-capsular injection of SH alleviates pain, improves functionality, and reduces joint crepitus [21,22,31]. Intra-articular administration of SH in degenerative disease normalizes synovial fluid viscoelasticity and activates tissue repair processes in the cartilage [23,24].

Based on this consideration, as proposed by other authors [32,33], the technique analyzed in our research used a combined approach in which the effect of the intracapsular administration of SH was added to the action of the arthrocentesis and the repetition of a cycle of 5 injections (1 per week). In the patient enrollment criteria for this study, we used both the DC/TMD classification system and the Wilkes staging system for TMJ internal derangement stages. Using both of these diagnostic systems allowed us to better define the clinical scenarios when performing this procedure is actually effective.

The analysis of our data showed an improvement in damage severity index at T1. The significant differences noticed (T0 *vs*. T1) demonstrate the efficacy of this treatment in the specific cases we treated. This evaluation is confirmed in TMJ imaging made at T1 where it showed increased thickness in both cartilage and disk and condylar excursion increase.

The analysis of the other indexes we assessed, furthermore, concur toward this positive evaluation. The increase of mouth opening capacity confirms the effects of the injected liquid pressure determining expansion of the intra-articular space and internal derangement improvement. The increase in masticatory efficiency shows the enhanced ability to chew harder food. Even the pain reduction is significant and appears to be linked to the decrease of inflammation and compression of the posterior part of the disc.

The functional and symptomatic improvement in our combined treatment, as shown by numerous researches [17,29,33,34], appears to be linked to:

1. Infiltration of the local anesthetic that is activated at the time of treatment;
2. Use of lavage solution, which removes the intra-articular catabolites;
3. Sodium hyaluronate infiltration, with its analgesic, anti-inflammatory, and lubricant properties.

The decrease of painful symptomatology at rest and the increase in masticatory efficiency and tolerability of the treatment determine quality of life improvement and the perceived good efficacy of the treatment by the patient.

## 5. Conclusions

The results of our study show that arthrocentesis with the addition of SH infiltration is a valid treating method for temporomandibular joint disorders. It has been demonstrated that the TMJ behaves similarly to other joints that orthopedic specialists commonly treat with SH, especially in terms of symptomatology and long-term functionality. Patients tolerate intra-articular infiltrations well, even though in some cases they may experience numbing of the facial nerve for the first few hours; this effect is linked with the use of local anesthetic.

The main objectives of our study were to evaluate the effectiveness of arthrocentesis and SH injection for patients with low and mild temporomandibular joint disorders. The data we collected are in accordance with literature and show improved mouth opening and decreased pain at rest and during mastication. These data were supported also by imaging: MRI showed mandibular condyle excursion increase and thickness increase in both cartilage and disk at T0 and T1. These features make arthrocentesis and sodium hyaluronate infiltration a valid treatment option. 

## Figures and Tables

**Table 1:** Demographic and clinical data at enrollment.

Characteristics		
Subjects	Total	28
Sex	Male, N (%) Female, N (%)	1 (3.5) 27 (96.5)
Age, mean (years)		40.6 (16–69)
Treatment tolerability (%)	Slight Moderate Good	7.2 46.4 46.4

**Table 2:** Clinical data at follow-up.

Indexes	T0	T1	*p* (T0 *vs*. T1)
Maximum non-assisted mouth opening, mean ± SD (millimeters)	36.3 ± 7.5	45.1 ± 1.9	<0.05
Severity joint damage, mean ± SD (Wilkes Score)	2.4 ± 0.9	0.4 ± 0.3	<0.05
Pain at rest, mean ± SD (VAS Score)	6.4 ± 2.5	0.7 ± 0.5	<0.0001
Pain during mastication, mean ± SD (VAS Score)	8.1 ± 1.7	0.9 ± 0.6	<0.0001
Mastication efficiency, mean ± SD (VAS Score)	3.1 ± 1.2	8.5 ± 1.2	<0.0001
